# Spontaneous perforation of a primary duodenal diverticulum stepped treatment model: A 10-patient case report

**DOI:** 10.3389/fsurg.2022.936492

**Published:** 2022-08-31

**Authors:** Bin Zhong, Zhonghu Li, Zhenyu Lin, Yanbing Shen, Jianxin Zhang, Weidong Jin

**Affiliations:** ^1^ The First School of Clinical Medicine, Southern Medical University, Guangzhou, China; ^2^ Department of General Surgery, Central Theater Command General Hospital of the Chinese People’s Liberation Army, Wuhan, China

**Keywords:** perforation, duodenum diverticulum, duodenum, stepped treatment, surgery

## Abstract

After colonic diverticula, a duodenal diverticulum (DD) is the second most common type of gastrointestinal diverticulum. DD is mainly caused by poor congenital development, resulting in a limited outward protrusion of the duodenal wall in a sac (primary diverticula). Perforation is one of the infrequent but most severe complications of DD, most commonly in the second segment of the duodenum (D2, 58%), followed by the third segment (D3, 30%). In the current case reports on the treatment of DD perforation, preoperative diagnosis is rare, with most patients being diagnosed and treated by laparotomy; the surgical approach is complex and varied, with artificial choices; and there is a high rate of complications and mortality (6%–34%) after surgical treatment. This study aimed to review our experience treating spontaneous perforation of the primary duodenal diverticulum, focusing on the surgical treatment model. A retrospective review of all spontaneous perforations of primary DD was conducted at one center between January 2010 and January 2022. We identified 10 patients with spontaneous perforation of primary DD (6 women and 4 men; median age: 51.5 years; range: 24–87 years). The patients had a median American Society of Anesthesiologists (ASA) score of 2. All patients underwent surgical treatment, of which six had percutaneous retroperitoneal drainage, two had diverticulectomy, one had distal gastrectomy + gastrojejunostomy + diverticuloplasty, and one had diverticulum repair. No patients died. The median length of stay was 12 days (range: 3–21 days). There were no long-term complications during the follow-up period (median follow-up of 12 months). A stepwise treatment model for spontaneous perforation of primary DD appears to have more advantages, and transabdominal exploratory surgery should probably not be the preferred treatment modality.

## Introduction

The diverticulum is round, oval, or tubular pouches that protrude from the gastrointestinal wall and can occur anywhere in the gastrointestinal tract. Duodenal diverticulum (DD) is the second most common type of gastrointestinal diverticulum after colonic diverticulum. The prevalence of duodenal diverticula in autopsies has been reported to be approximately 22% (1).

Perforation is a rare but severe complication of duodenal diverticula (2). Clinically, most DD perforations occur in the retroperitoneal position, resulting in a lack of characteristic presentation and abdominal signs, and the diagnosis is often delayed (1). At the same time, leakage of digestive fluid and the formation of retroperitoneal abscesses make it difficult for the infection to resolve on its own, often requiring surgical treatment. Laparotomy is still considered necessary in hemodynamic instability patients and patients with sepsis, especially if the diagnosis is unclear (3, 4). Despite the duodenum having gastric juice, bile, pancreatic juice, intestinal juice, and other digestive solutions, the lack of surrounding tissues to aid closure of the perforation and the edema of the intestinal wall around the perforation results in a low success rate of perforation repair.

In contrast, relatively radical procedures (e.g., gastrectomy, diverticulectomy, pancreaticoduodenectomy, etc.) are more invasive and highly risky in emergencies. At the same time, the surgical approach has a considerable impact on the patient's physiological function. Laparotomy disrupts the retroperitoneum, a vital defense barrier of the organism, which may result in postoperative abdominal infection and drainage that cannot be easily completed. This study aimed to review our 12-year experience treating spontaneous perforation of primary DD in a single center, focusing on surgical treatment, to explore an appropriate treatment modality for DD perforation.

## Case report

Between January 2010 and January 2022, all perforated DD cases were retrospectively reviewed at the Department of General Surgery, Central Theater Command General Hospital of the Chinese People's Liberation Army. Perforations due to trauma and Iatrogenic perforations (e.g., during endoscopy) were excluded from the study. We identified 10 patients with spontaneous perforation of primary DD (6 women and 4 men; median age: 51.5 years; range: 24–87 years). The patients had a median American Society of Anesthesiologists (ASA) score of 2 (range: 2–3). All patients underwent surgical treatment, of which six had percutaneous retroperitoneal drainage, two had diverticulectomy + drainage, one had distal gastrectomy + gastrojejunostomy + diverticuloplasty, and one had diverticulum repair + retroperitoneal drainage (Table 1). All patients underwent CT scans preoperatively.

**Table 1:** Patient characteristics, treatment modalities, and prognosis.

Case	Age (yr)	Sex	ASA	Shock	Cardinal symptoms	Peritoneal irritation sign	History of abdominal surgery	Diagnosis	Perforation localization	Resume oral feeding (d)
1	77	Woman	2	No	Acute epigastria pain	Yes	No	CT	D2	6
2	48	Woman	3	No	Recurrent epigastric pain	No	Yes	Surgery	D2	7
3	65	Woman	2	No	Acute epigastria pain	No	No	Surgery	D2	5
4	26	Woman	2	No	Acute epigastria pain	Yes	Yes	Gastrointestinal imaging	D3	12
5	60	Man	2	No	Severe nausea and vomiting	No	Yes	CT	D2	10
6	87	Woman	2	No	Acute abdominal pain	Yes	No	Gastrointestinal imaging	D2	4
7	24	Man	2	No	High fever	No	No	Surgery	D2	Unknow
8	26	Man	2	No	Right lumbar pain	No	No	Surgery	D2	5
9	45	Man	2	No	Acute epigastria pain	Yes	No	Surgery	D2	6
10	55	Woman	3	No	Upper abdominal pain	No	Yes	Surgery	D3	10
Case	Surgery	Double driving pipes placement	Nasogastrointestinal tube	Morbidity-mortality	Hospital stay (d)	Follow-up	Shock	Perforation localization	Resume oral feeding (d)	
1	Diverticulectomy + drainage	Yes	Yes		14	Lost after 24 month of follow-up	No	D2	6	
2	Distal gastrectomy+gastrojejunostomy+diverticuloplasty	No	Yes		13	Lost after 12 month of follow-up	No	D2	7	
3	Diverticulectomy+drainage	Yes	Yes		8	Lost after 18 month of follow-up	No	D2	5	
4	Percutaneous retroperitoneal drainage	Yes	Yes		21	Lost after 12 month of follow-up	No	D3	12	
5	Percutaneous retroperitoneal drainage	No	Yes		17	Lost after 8 month of follow-up	No	D2	10	
6	Percutaneous retroperitoneal drainage	Yes	Yes		9	Lost after 12 month of follow-up	No	D2	4	
7	Percutaneous retroperitoneal drainage	Yes	Yes	Unknow	3	Lost after discharge	No	D2	Unknow	
8	Percutaneous retroperitoneal drainage	Yes	Yes	Incisional infection	9	Lost after 6 month of follow-up	No	D2	5	
9	Diverticulum repair+retroperitoneal drainage	Yes	Yes		11	Lost after 12 month of follow-up	No	D2	6	
10	Percutaneous retroperitoneal drainage	Yes	Yes		15	Lost after 16 month of follow-up	No	D3	10	

ASA, American Society of Anesthesiologists.

**Figure 1:**
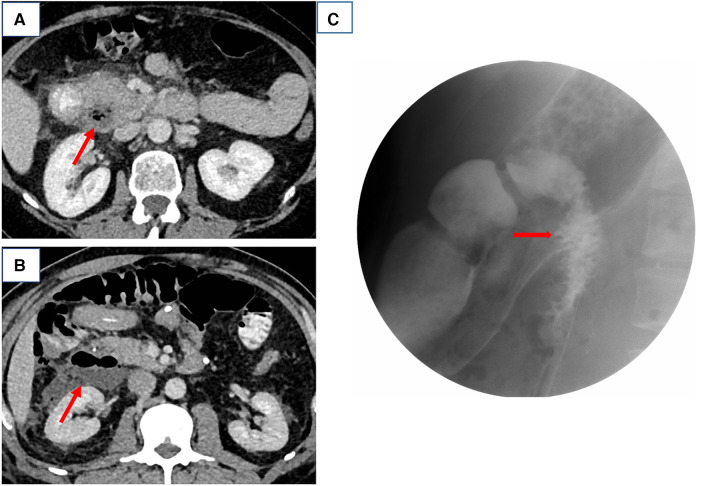
(**A,B**) Computed tomography scans of cases 5 and 1 and (**C**) gastrointestinal barium study of case 4, both suggestive of a perforated duodenal diverticulum.

We here report 10 cases of spontaneous DD perforation (Table 1). The clinical presentation was abdominal pain without fever in five cases, abdominal pain with fever in three cases, right-sided lumbar pain in one case, and vomiting in one case. Seven patients had elevated white blood cell count and C-reactive protein, two patients had white blood cell count and C-reactive protein in the normal range, and one patient had a decreased white blood cell count. None of the patients had a severe infectious shock on admission. Of note, one patient was admitted with a high fever (maximum temperature 40.8°C) without signs of peritoneal irritation, two patients had localized peritoneal irritation signs, and one patient presented with the peritoneal irritation sign throughout the abdomen. In total, 20% (2/10) of the cases could be diagnosed as duodenal diverticulum perforation by CT (Figures 1A,B). DD perforation can be diagnosed by gastrointestinal imaging in 20% (2/10) of the cases (Figure 1C). The rest of the cases were confirmed by intraoperative diagnosis. Eight of these cases had an ASA score of 2, and two had an ASA score of 3. The perforated DD was located in the D2 level in eight cases (80%) and the D3 level in two cases (20%). All patients received postoperative intravenous antibiotic therapy (cefoperazone/sulbactam) for 5 d.

In 2 of the 10 patients, no double driving pipes were placed intraoperatively for continuous postoperative flushing and drainage. In all patients, a nasogastric or nasojejunal tube (1:1) was placed to reduce postoperative bowel tension and facilitate enteral nutrition support. Four of the ten patients had a history of previous abdominal surgery. One patient had a previous diagnosis of adhesive intestinal obstruction and underwent intestinal adhesion lysis, after which he continued to have recurrent abdominal pain for a long time. This admission CT revealed thickening of the gastric wall at the gastric sinus and a diverticulum of the descending duodenum and surrounding exudate. In combination with the patient's previous history of mild vomiting, the patient was considered to have pyloric stenosis. Therefore, a distal gastrectomy + Roux-n-Y anastomosis + duodenal exploratory was planned using the previous median incision. Intraoperative exploration revealed a diverticulum located in the posterior wall of the descending duodenum with a 0.1 cm diverticular rupture, so additional duodenal diverticuloplasty was performed. No double driving pipes were placed.

We considered that with the retroperitoneal barrier intact, the inflammatory exudate due to infection, although showing a gradual increase, was generally limited and did not affect the systemic situation as much as expected. In all six patients, we made a small vertical incision of approximately 5 cm in the right mid-axillary line at the level of the umbilicus for debridement and drainage of the retroperitoneal abscess under ultrasound guidance, which protected the peritoneum, a critical defense barrier of the body, and was less likely to lead to postoperative infection of the peritoneal cavity.

**Figure 2:**
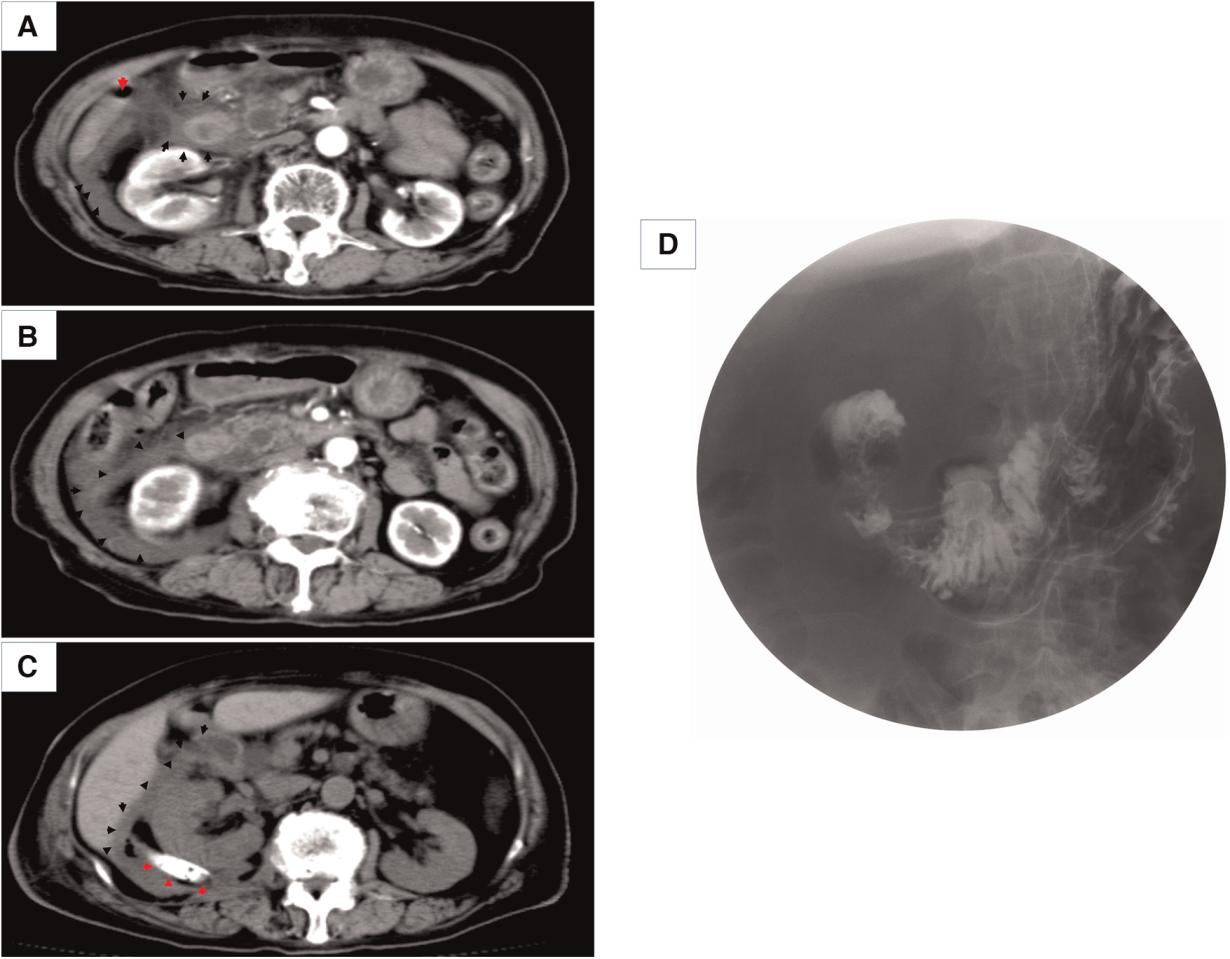
(**A,B**) Enhanced CT scans of the patient on admission and 7 days after conservative treatment (red arrows point to free abdominal gas, black arrows point to peri-duodenal and retroperitoneal exudate). (**C**) CT scan 7 days after percutaneous retroperitoneal tube placement for drainage (red arrows point to the placed retroperitoneal double cannula, black arrows point to periduodenal and retroperitoneal exudate). (**D**) shows a gastrointestinal barium study of the patient.

**Figure 3:**
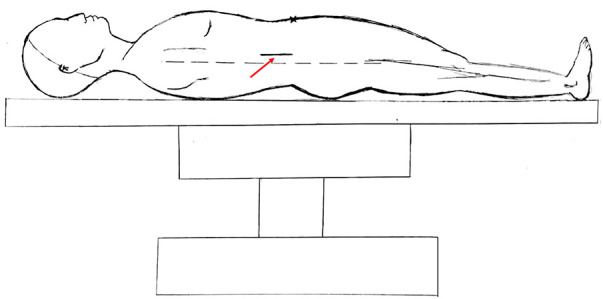
Hand drawing of retroperitoneal abscess drainage through a small incision in the right lumbar region. The red arrow points to the location of the incision, approximately 3–5 cm. the dotted line is the mid-axillary line.

A typical case is described in detail here. The patient was an 87-year-old woman admitted to the hospital with a sudden onset of epigastric pain for 8 h. On admission, her vital signs were stable. On examination, she was found to have mid-upper abdominal pressure and suspicious rebound pain, and the rest of her abdomen was free from peritoneal Irritation signs. Laboratory investigations revealed the following: WBC 5.8*10^9^/L, NP 73.2%, PLT 87*10^9^/L; AMY 1,450U/L, TB 34.90 µmol/L, CB 19.00 µmol/L, ALT 492U/L, and AST 515U/L. The CECT scan suggested (Figure 2A) suspected duodenal diverticulum perforation. Given the patient's stable vital signs and the absence of significant peritoneal irritation, but with advanced age and hepatic insufficiency, there was no better surgical access on the CT scan. We decided to treat the patient with nonoperative management for one week (anti-infective, fasting, gastrointestinal decompression, and maintaining acid–base balance). The patient's blood count and liver and kidney function were dynamically reviewed during conservative treatment. During this period, the patient's white blood cells gradually increased, and she developed intermittent fever with a maximum temperature of 38.5°C. One week later, a repeat CECT revealed a significant increase in fluid accumulation around the lateral descending duodenum, posterior ascending colon, and perirenal fat sac (Figure 2B). Gastrointestinal imaging showed that the diverticulum was located at the beginning of the horizontal segment of the duodenum, and contrast leakage was seen, leading to a definite diagnosis of retroperitoneal perforation of the duodenal diverticulum (Figure 2D). We then performed percutaneous retroperitoneal drainage through a small incision in the right lumbar region under basis anesthesia and local anesthesia with ultrasound localization (Figure 3). The patient's temperature and inflammatory indicators (white blood cell count, C-reactive protein, etc.) gradually returned to normal levels after the operation. A review of CT scans on postoperative day 7 showed a significant reduction in retroperitoneal fluid (Figure 2C), and the patient was discharged 9 days after surgery.

Only one patient developed a postoperative complication of incisional infection after surgery. The remaining patient had no long-term complications during the postoperative period and postdischarge follow-up (median follow-up of 12 months). The median length of stay was 12 days (range: 3–21 days). One patient considered himself to be recovering well on the third postoperative day and strongly requested to be discharged. There were no long-term complications during the follow-up period (median follow-up of 12 months). The case characteristics, treatment modalities, and prognosis of the 10 patients are placed in Table 1.

## Discussion

DD was first described by Chomel in 1710 (5). Since then, scientists have continued to publish reports related to DD (6, 7). DD perforation is rare in the acute abdomen and is often difficult to distinguish from duodenal perforation, which is usually associated with diverticulitis or ischemia due to distention of food within the diverticulum (8). Basically, the causes of perforation are multiple and include diverticulitis, enterolithiasis, ulceration, iatrogenic perforation during ERCP sphincterotomy, trauma, foreign body, and so on (9). Farné et al. (10) recently reviewed the available literature and now reported 210 cases of DD perforation. The preoperative diagnosis and treatment choice are the two issues that currently need to be addressed for DD perforation. The symptoms of DD perforation are often ill-defined from the available case reports. The most common clinical presentation appears to be right upper abdominal pain, as reported in this article. Although DD perforation may have the same presentation on CT as duodenal perforation, there is no denying that the number of cases of preoperative diagnosis of DD perforation has improved since physicians have commonly used CT. We believe that CT/enhanced CT will be the primary diagnostic method for DD perforation in the future. In our clinical work, we do not routinely use oral contrast. Very rarely, we use it when we do not clearly see free intraperitoneal air on the CT scan and there is a persistent suspicion of perforation, but it does not seem to impact our treatment. This is somewhat consistent with the WSES guidelines (11). This test may improve the diagnostic accuracy of duodenal diverticulum perforation, which still needs to be validated in higher quality and multicenter studies. Of course, whether such tests can lead to disease changes and inconvenience to treatment needs further explored.

**Figure 4:**
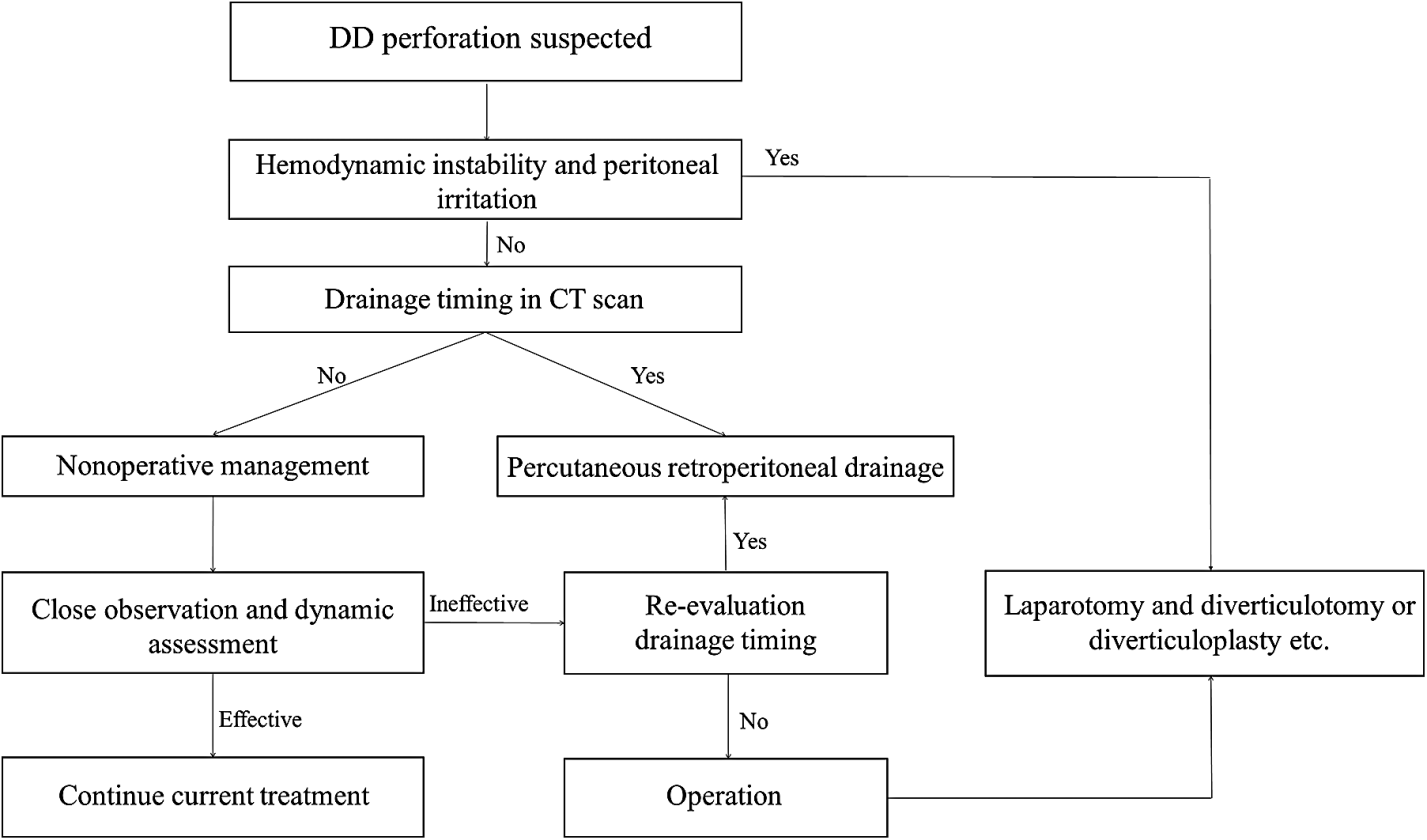
Flow diagram of step-up treatment model for reference.

Due to the small number of cases, there are no relevant guidelines for managing perforated duodenal diverticula. Although there are reports of surgical treatment, conservative treatment, and intermediate treatment, a review of these reports shows that there is still no treatment model available to physicians in clinical practice for these patients. The choice of treatment is a function of the surgeon's experience and control of the condition (7). It has been suggested in the literature that the looseness of the retroperitoneal tissue space and the large size of the retroperitoneal space make it extremely dangerous for infection to spread (12). However, a review of our cases shows that most infections are not very dangerous when duodenal diverticula leads to perforation of the posterior wall; with the posterior peritoneal barrier intact, the infected exudate, although progressively increasing, is generally limited and does not have as significant an impact on the general condition as expected. If the patient is in poor general condition, hemodynamically unstable, or septic, surgical treatment is associated with a high risk. Especially when faced with emergency surgery, even a simple diverticulectomy can be difficult for the surgeon (13). Exploratory laparotomy should probably not be the first choice of treatment. We have developed a step-up treatment model for this purpose for reference (Figure 4). In suspected retroperitoneal perforation of the duodenal diverticulum, nonoperative management (anti-infection, fasting, nutritional support, decompression, etc.) is indicated. In the application of antibiotics, we commonly use drugs that cover Gram-negative bacterium and/or anaerobic organisms (e.g., cefoperazone/sulbactam + metronidazole). We do not usually use antifungals routinely, except when antibiotic therapy is not effective and fungal infection is highly suspected and when a fungal culture of the peritoneal fluid is performed, and based on the culture results, the relevant departments (Infection and Pharmacology) are consulted. The need for antifungal treatment is discussed. This is because fungal cultures (+) often have the potential for false positives [due to improper sampling or specimen contamination) and the effectiveness of antifungal therapy in patients with fungal cultures (+) is currently unclear and controversial (14].

When conservative treatment is not well controlled, percutaneous retroperitoneal drainage is selected at the right time, and when the patient shows clear signs of peritonitis, open surgery is performed. Diverticulectomy is the major and most common surgical method; it is suggested when conservative treatment fails or indicates a massive hemorrhage or failure of endoscopic hemostasis. However, simple suture closure of the duodenal wall after diverticulectomy is reported to be associated with a risk of duodenal fistula, which carries a mortality of 20%–30% (15). Sometimes diverticulectomy is associated with a biliodigestive bypass to reduce the risk of duodenal leak or fistula and to reduce morbidity. Therefore, we prefer conservative treatment and percutaneous retroperitoneal drainage after repeated revaluation. For percutaneous drainage timing, we can determine the extent of the infection by CT scan. The better time to operate is when the abscess extends posteriorly to the level of the right margin of the ascending colon. At this point, there are several advantages to placing a small incision in the lateral lumbar region for drainage by ultrasound. First, the abscess is closest to the body surface at this time, which has little impact on the patient, and the operation can be completed under basis anesthesia plus local anesthesia. Second, access to the retroperitoneal space is convenient and straightforward at this time, making the operation relatively simple and less likely to injure other organs. Third, the integrity of the retroperitoneal barrier is maintained so that infection and digestive fluid exudation are confined to the retroperitoneal space without contaminating the abdominal cavity, and drainage is complete. We did not study the relationship between the size of perforation and the selection of treatment. However, we believe that the location (the portion or wall of the duodenum) of the perforation also plays a vital role in this question.

This article presents one of the most series of cases published to date (10 patients). The overall results were satisfactory, and no patient died. Recent reviews have reported a mortality rate of approximately 16.7% (13). Our results compare favorably with this. However, as the number of cases is still small, our treatment model needs to be validated with many cases. With the rapid development of endoscopic techniques in recent years, many previously required surgical treatment problems can now be addressed endoscopically. There are reports in the literature that endoscopic drainage of the perforation by endoscopy may be a minimally invasive alternative to percutaneous drainage or exploratory laparotomy when CT scans suggest that the effusion is confined in clinically stable patients to the retroperitoneal space (16, 17). Shirobe et al. (17) and Fan et al. (16) have endoscopically placed drains through the diverticulum to place a drainage tube, while Sasaki et al. (18) used polyglycolic acid sheets and fibrin glue to treat the perforation with endoscopic tissue shielding, and they both achieved good results. Future treatment trends will undoubtedly be toward more accessible application, less invasive, and better prognosis. We need to continue exploring and validating new treatment modalities in clinical practice to advance the cause of medicine.

## Conclusion

Surgical treatment by exploratory laparotomy has long been the method of choice for most surgeons when the diagnosis is unknown. However, with advances in medical technology and imaging techniques, less invasive methods are available, and laparotomy surgical treatment may not be the first option. We aim to explore a more rational surgical treatment model by retrospectively reviewing 10 previous cases of spontaneous perforation of primary DD . We suggest a step-up treatment strategy. The clinician may choose the appropriate treatment, either conservative or surgical, depending on the different clinical conditions and thus on the outcomes.

## Data Availability

The original contributions presented in the study are included in the article/Supplementary Material; further inquiries can be directed to the corresponding author.
